# Editorial: The molecular basis of programmed cell death and neuroinflammation in neurodegenerative diseases

**DOI:** 10.3389/fimmu.2023.1359136

**Published:** 2024-01-11

**Authors:** Chetna Soni, Antariksh Tyagi, Xusheng Wang, Ramkumar Mathur

**Affiliations:** ^1^ Department of Pathology, Grossman School of Medicine, New York University, New York, NY, United States; ^2^ Yale Center for Genome Analysis, Yale School of Medicine, West Haven, CT, United States; ^3^ Department of Genetics, Genomics & Informatics, University of Tennessee Health Science Center, Memphis, TN, United States; ^4^ Department of Geriatrics, School of Medicine and Health Sciences, University of North Dakota, Grand Forks, ND, United States

**Keywords:** neuroinflammation, program cell death, neurodegenerative diseases, apoptosis, necroptosis

The pathogenesis of neurodegenerative diseases is closely linked to the occurrence of programmed cell death, also known as apoptosis and neuroinflammation. Despite the presence of a distinct mode of cell death exhibiting unique morphological characteristics, the identification and implications of this phenomenon in neurological diseases remain unknown. Current scientific investigations are diligently striving to identify and understand distinct cellular and molecular targets that can be selectively engaged in therapeutic intervention for Alzheimer’s disease (AD), Parkinson’s disease (PD), and Huntington’s disease (HD). This special edition is dedicated to a diverse array of original research, comprehensive literature reviews, and expert opinions that its aim is to provide a deeper understanding of the intricate molecular mechanisms underlying programmed cell death in the context of neurodegenerative disorders.

The perspective article from Zhang et al. presents a comprehensive analysis of the landscape of necroptosis research spanning the period from 2012 to 2021. The investigation of necroptosis has exhibited a sustained and consistent expansion throughout the previous decade. The current focus of research revolves around the phenomenon of synergistic interactions with ferroptosis, with an increasing emphasis on the exploration of RIPK1/RIPK3/MLKL pathways. This line of investigation aims to elucidate the intricate connections between ferroptosis, inflammation, and oxidative stress while also exploring the translational applications and therapeutic prospects for combating cancer and neurological disorders.

The seminal study conducted by Lee et al. introduced a chronic constriction injury (CCI) model as an effective animal model for investigating neuropathic pain. Their research further demonstrated that the administration of Mesenchymal stem cells (MSCs) in spheroid form exhibited enhanced efficacy in promoting pain resolution and cell viability when compared to monolayer cells. In this study, the researchers observed that MSC spheroids consisting of 10,000 cells displayed enhanced resistance to apoptosis, a programmed cell death process, and exhibited decreased secretion of immune response factors after CCI. This study presents compelling evidence supporting the notion that spheroids possess the capacity to enhance the efficacy of MSC-based therapeutic interventions targeting induced neuropathic pain.

The review article authored by Li et al. provides an analysis of the intricate metabolic and regulatory pathways associated with tryptophan (TRP). A substantial body of preclinical research has been conducted to investigate the modulation of TRP metabolism as a potential therapeutic approach for neurological and psychiatric disorders. However, the majority of these studies have primarily concentrated on examining the involvement of 5-HT, TRP, KYN metabolites, and IDO expression, while paying limited attention to the intricate interplay of complex enzymes. The investigation of enzymes involved in TRP metabolism necessitates the utilization of molecular, cellular, and tissue methodologies, as they serve as crucial regulators within the TRP pathway.

The current knowledge regarding the involvement of the central nervous system (CNS) and peripheral inflammatory changes in Huntington’s disease (HD) remains limited. In the comprehensive review, Jia et al. provide a concise synthesis of current scientific literature about immune and inflammatory changes observed in individuals with HD. This study encompasses investigations conducted on animal models of HD as well as clinical observations of patients diagnosed with HD. The investigation into the specific impact of inflammation on the development of Huntington’s disease has promising implications for the development of innovative treatment approaches.

Genomic instability is a key driver of various neurodegenerative disorders and central nervous system malignancies. The comprehensive review by Suptela et al. presents the occurrence of cytosolic DNA sensors in resident central nervous system (CNS) cells and their capacity to actively modulate their reactions to self-DNA. In this study, the author discusses how glial DNA sensors can prevent cancer and neuroinflammation, which are known to cause neurodegenerative diseases. The processes by which glia detect cytosolic DNA and the relative involvement of each pathway at different stages of CNS diseases will help us understand their pathophysiology while developing new treatments.

The growing study has shown chlorogenic acid’s anti-inflammatory and immunomodulatory effects. In this seminal research article, Xiong et al. elucidate the molecular mechanisms and specific targets associated with the potential therapeutic effects of chlorogenic acid on neuroinflammation. The present study demonstrates the notable inhibitory impact of Chlorogenic acid on the polarization of microglia towards the M1 phenotype. Furthermore, it amplifies the cognitive impairment induced by neuroinflammation in a murine model.

The Lipopolysaccharide (LPS)-induced depression-like model in mice is a widely employed approach for investigating the underlying mechanisms of depression associated with inflammation as well as evaluating the potential therapeutic benefits of various drugs. Yin et al. conducted a thorough systematic review aimed at identifying appropriate animal models for future investigations on inflammation-related depression and highlighted the significant influence of mouse strains and lipopolysaccharide (LPS) administration on the assessment of behavioral outcomes in relevant experimental models.

Levodopa (L-DOPA) is frequently administered to patients with Parkinson’s disease (PD) by healthcare providers in clinical settings to improve their symptoms. Despite long-term administration of L-DOPA, a significant proportion of patients experience various complications, including the “on-off phenomenon,” decreased therapeutic efficacy, and levodopa-induced dyskinesia (LID). The review by Zhang et al. examines the effects of widely used anti-inflammatory medicines and NMDA receptor antagonists on central nervous system neuroinflammation and LID progression. This study presents a novel theoretical framework for the identification of potential therapeutic targets for LID.

Finally, in a recent study, the therapeutic potential of Olfactory ensheathing cells (OECs), a specific type of glial cell, has been demonstrated in the treatment of various neurological disorders. OECs have been shown to possess therapeutic potential for the treatment of neurological disorders. In their comprehensive review, Zhang et al. highlight the significance of OECs as a promising therapeutic approach for various nervous system diseases.

Overall, the Research Topics focus on novel and impactful investigations into the mechanisms of neuronal cell death and the activation of microglia. These processes are known to play a crucial role in the development of neuroinflammation and neurodegenerative disorders. The insights gained from this Research Topic will certainly improve our understanding of the consequences of programmed cell death and the molecular mechanisms involved in mitigating neurodegenerative diseases.

## Author contributions

CS: Conceptualization, Data curation, Formal analysis, Investigation, Methodology, Project administration, Software, Supervision, Validation, Writing – review & editing. AT: Conceptualization, Data curation, Formal analysis, Investigation, Methodology, Project administration, Software, Supervision, Validation, Visualization, Writing – review & editing. XW: Conceptualization, Data curation, Formal analysis, Investigation, Methodology, Project administration, Software, Supervision, Validation, Writing – original draft, Writing – review & editing. RM: Conceptualization, Data curation, Formal analysis, Funding acquisition, Investigation, Methodology, Project administration, Resources, Software, Supervision, Validation, Visualization, Writing – original draft, Writing – review & editing.

